# Gastrointestinal Vagal Afferents and Food Intake: Relevance of Circadian Rhythms

**DOI:** 10.3390/nu13030844

**Published:** 2021-03-05

**Authors:** Amanda J. Page

**Affiliations:** 1 Adelaide Medical School, University of Adelaide, Adelaide, SA 5000, Australia; amanda.page@adelaide.edu.au; Tel.: +61-8-8128-4840; 2 Nutrition, Diabetes and Gut Health, Lifelong Health Theme, South Australian Health and Medical Research Institution (SAHMRI), Adelaide, SA 5000, Australia

**Keywords:** vagal afferents, circadian, food intake, gastrointestinal tract

## Abstract

Gastrointestinal vagal afferents (VAs) play an important role in food intake regulation, providing the brain with information on the amount and nutrient composition of a meal. This is processed, eventually leading to meal termination. The response of gastric VAs, to food-related stimuli, is under circadian control and fluctuates depending on the time of day. These rhythms are highly correlated with meal size, with a nadir in VA sensitivity and increase in meal size during the dark phase and a peak in sensitivity and decrease in meal size during the light phase in mice. These rhythms are disrupted in diet-induced obesity and simulated shift work conditions and associated with disrupted food intake patterns. In diet-induced obesity the dampened responses during the light phase are not simply reversed by reverting back to a normal diet. However, time restricted feeding prevents loss of diurnal rhythms in VA signalling in high fat diet-fed mice and, therefore, provides a potential strategy to reset diurnal rhythms in VA signalling to a pre-obese phenotype. This review discusses the role of the circadian system in the regulation of gastrointestinal VA signals and the impact of factors, such as diet-induced obesity and shift work, on these rhythms.

## 1. Introduction

In response to a meal, the gastrointestinal tract sends humoral and neural signals to the central nervous system where it is combined with other signals ultimately leading to the termination of a meal. These satiety signals are not static, displaying a high degree of plasticity [1]. For example, gastric vagal afferents (VAs) display diurnal rhythms in their response to food related stimuli [2]. These rhythms are inversely associated with meal size and meal frequency and thus stomach content [2,3], such that in mice the nadir in gastric VA sensitivity during the dark phase is associated with an increase meal size and meal frequency, whereas the peak in sensitivity during the light phase is associated with reduced meal size and meal frequency [3]. This is important for the fine regulation of food intake to meet the daily fluctuations in energy demand and forms a component of the circadian systems role in energy homeostasis, namely temporal regulation of appetite and food intake as well as metabolic processes, such as anabolism and catabolism [4].

Circadian rhythms occur over ~24 h and are driven by endogenous clocks that form a sequence of interlocking molecular loops. The master clock is located in the suprachiasmatic nucleus (SCN) and receives input from the retina to allow entrainment to the light-dark cycle [5]. However, there are other clocks located both centrally and peripherally. For example, VA neurons express clock gene molecules which oscillate over a 24 h period [2]. Disruption of these VA rhythms, such as occurs in diet-induced obesity or shift work conditions, could lead to disruption in the timing of food intake contributing to the misalignment of metabolic processes and perpetuating the issue.

This review focusses on current knowledge on gastrointestinal VA satiety signalling, with particular emphasis on the diurnal regulation of these signals and the potential significance of these rhythms in the fine control of food intake. In addition, the impact of disrupted satiety signalling on food intake patterns and the development/maintenance of obesity will be discussed as well as the potential benefits of time restricted feeding.

## 2. Gastrointestinal Vagal Afferents

### 2.1. Subtypes of Gastrointestinal Vagal Afferents

Gastrointestinal VAs play an important role in food intake regulation [6], signalling to the hindbrain the arrival, amount and chemical composition of a meal. This information is then processed leading to reflex control of gut motility and secretions, required for the digestion and absorption of a meal, and sensations of satiety and fullness, that ultimately terminate a meal. Therefore, gastrointestinal VAs play an important role in limiting food intake and the size of a meal. Gastrointestinal VAs can be classified, based on their response to various stimuli, into three main groups, namely mechano-, chemo- and thermoreceptors, all with specific roles in gastrointestinal physiology, however, this review will focus on mechano- and chemoreceptors due to their known or suspected role in food intake regulation [7].

#### 2.1.1. Mechanoreceptors

**Figure 1:**
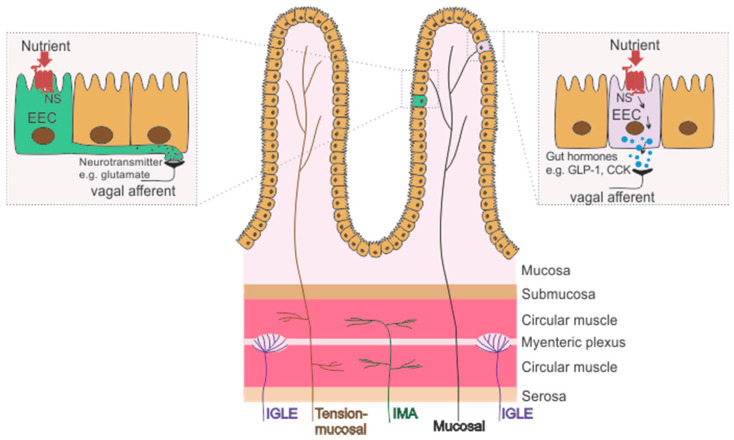
Schematic of the wall of the gastrointestinal tract with the location of the receptive fields of subclasses of gastrointestinal vagal afferents (VAs), including mechanosensitive (mucosal stroking) and/or chemosensitive mucosal afferents (Black), intraganglionic laminar endings (IGLEs, tension receptors; Purple), stretch sensitive intramuscular arrays (IMAs, stretch receptors; Green) and tension-mucosal afferents (Brown; sensitive to mucosal stroking and stretch). Chemosensing occurs via specialized enteroendocrine cells (EECs) that express nutrient receptors (NS) which when activated initiate an intracellular process culminating in: (1) the release of gut hormones, such as cholecystokinin (CCK) and glucagon-like peptide 1 (GLP-1) which subsequently act on peripheral VA terminals; or (2) the release of a neurotransmitter, such as glutamate, directly onto VA endings via neuropods that protrude from the basolateral surface of EECs.

Mechanosensitive gastrointestinal VAs are important for sensing the amount and movement of food as it passes through the gastrointestinal tract. The receptive fields for mechanosensitive VAs are located within the mucosal and muscular layers of the gut wall [8] (Figure 1), and are categorised into tension, stretch, mucosal and tension-mucosal receptors depending on their response to specific mechanical stimuli [9,10]. These different types of afferent are discussed below:

##### Tension or Stretch Receptors

Tension receptors are generally slowly adapting, low threshold mechanoreceptors that respond to circular tension [11,12]. Tension receptors are thought to have specialized endings with a flattened leaf-like structure surrounding the myenteric ganglia termed intraganglionic laminar endings (IGLEs) [13,14]. Recently, a subpopulation of gastric IGLEs, which express glucagon-like peptide 1 receptor (GLP-1R), was activated by mechanical distension in vivo in mice [15,16]. In addition, there is a population of IGLEs in the small intestine, which express the oxytocin receptor gene (*Oxtr*) [15], which when activated by optogenetic and chemogenetic stimulation dramatically reduced food intake [15]. This suggests there are small intestinal mechanosensitive afferents which contribute to the regulation of food intake.

Gastrointestinal VA tension receptors were generally thought to be a homogenous population of afferents that detect both muscular tension and stretch [9,17]. However, stretch and tension are two completely different forces with tension the force required to maintain muscle length and stretch reflecting the need for muscle extension or contraction [9]. The identification of two morphologically distinct VA ending in the muscle layer of the gut raised the possibility of two distinct populations of stretch and tension receptors [18], with intramuscular arrays proposed as stretch receptors [9]. Intramuscular arrays are VA endings that run in parallel to muscle bundles in the muscularis externa [18,19,20].

##### Mucosal Receptors

Mucosal mechanoreceptors innervate the mucosal layer of the gastrointestinal tract and are fast adapting, low threshold mechanoreceptors activated by mucosal stroking, such as occurs when food particles pass over the receptive field. Mucosal receptors are relatively uninvestigated but, in the stomach, these receptors are thought to be involved in the control of gastric emptying, through the detection of food particle size, as well as the vomiting reflex [7,9,21,22], however, there is no direct in vivo evidence that this is the case.

##### Tension-Mucosal Receptors

Tension-mucosal receptors have been observed in the ferret oesophagus and respond to both circular tension and mucosal stroking [12]. However, using a similar approach a distinct population of tension-mucosal receptors could not be identified in the mouse [11]. This is possibly a consequence of the thinness of the oesophageal tissue in the mice, where low intensity mucosal stroking with von Frey hairs (e.g., 10 mg) can also stretch the underlying muscular layer making it impossible to distinguish between tension and tension-mucosal receptors [11]. Nonetheless, soon after their identification a similar subpopulation of colonic splanchnic and pelvic afferents, termed mucosal-muscular receptors, were identified in mice [23]. It has been suggested that these afferents have multiple endings that terminate in the muscular and mucosal layers of the gut wall [12,24]. However, although there is evidence that a single dorsal root ganglia neuron can receive input from a number of endings within the gut wall [25,26], including the mucosa, myenteric ganglia and circular muscle, the location of vagal tension-mucosal afferents remains to be determined and it is possible that a single ending in the subepithelial plexus is responsive to both stretch and mucosal stroking [10].

#### 2.1.2. Chemoreceptors

Gastrointestinal VA chemoreceptors, located in the mucosal lamina propria of the gut wall, detect a wide range of stimuli, including changes in pH, osmolarity, gut hormones and nutrients [27] (Figure 1). While some chemosensitive afferents respond directly to nutrients, such as glucose [28], mucosal VAs do not make direct contact with the luminal content and instead chemosensing mechanisms are mediated by specialized epithelial cells in the gut wall, known as enteroendocrine cells (EECs), which express gut hormones. Different EECs express different specialized nutrient receptors with meal consumption and digestion resulting in a complex pattern of gut hormone release [29]. These hormones then act on their receptors on VA endings which signal to the brain and initiate the termination of food intake [30]. For example, glucose in the lumen of the small intestine induces release of the gut hormones glucagon-like peptide 1 (GLP-1) and 5-hydroxytryptamine (5-HT), which subsequently activate VA endings in the intestinal mucosa ultimately contributing to the regulation of gut motility (e.g., gastric emptying) and intestinal fluid secretion [30]. In addition, fatty acids and amino acids have been shown to induce cholecystokinin (CCK) release which subsequently activates VAs to induce satiety [31]. Further, VAs, in the distal intestine, have been shown to make synaptic connections with EECs via axon-like projections, known as neuropods, that protrude from the basolateral surface of EECs [32,33,34,35,36]. It has been demonstrated that infusion of sucrose evoked VA firing, through the release of glutamate from the neuropod onto the VA ending [37].

### 2.2. Gastrointestinal Vagal Afferents and Food Intake Regulation

Gastrointestinal VAs are important for the short term regulation of food intake and meal size. To date most of our knowledge relates to the role of VA mechanosensitive tension/stretch receptors and chemoreceptors and, therefore, these receptors will be the focus of this review. As food moves through the gastrointestinal tract the VA mechano- and chemoreceptor signalling occurs in a coordinated manner as outlined below:

#### 2.2.1. Gastric Signals

As food enters and gradually fills the stomach it causes distension of the stomach wall which activates tension or stretch receptors innervating the stomach wall. These are one of the first signals to induce feelings of fullness and satiety [38,39,40,41]. The distension component of a meal can be reproduced in humans and separated from the nutrient component using a bag. Inflating a bag with air in the proximal stomach reduced hunger and induced a sensation of pressure-like fullness [38,40]. Distension of the proximal stomach by filling the bag with water induced feelings of fullness [42] and filling the bag with 400–800 mL of water reduced food intake in a volume-dependent manner [43]. Further, gastric distension, before or during a meal, reduced food intake in humans [41]. The antrum has also been shown to play a role in the perception of fullness and termination of a meal and it has been shown that fullness is directly related to the volume of a 350 mL glucose test drink in the distal stomach [44]. Further, after a mixed-nutrient drink subsequent energy intake was inversely associated with antral volume prior to the meal [45]. 

In a recent mouse study, Bai et al. showed that a subset of GLP-1R positive neurons are gastric IGLE mechanosensitive tension receptors [15]. Further, optogenetic and chemogenetic activation of these GLP-1R positive neurons inhibited food intake, albeit not to the same degree as activation of oxytocin receptor positive neurons which are predominantly small intestinal IGLE mechanosensitive tension receptors [15]. Nonetheless, it is clear that activation of tension receptors in the stomach initiates the satiety signalling. 

#### 2.2.2. Small Intestinal Signals

As gastric emptying occurs chyme enters the small intestine and the gastric distension signals diminish to be replaced by small intestinal signals. The intestine is the major site of macronutrient breakdown and nutrient absorption. However, the small intestine is not just the site of nutrient absorption but also nutrient detection with extensive sensory VA innervation of the small intestine, with peak density in the duodenum [10,46]. Recent evidence indicates mechanosensitive tension receptors in the duodenum play a more important role in satiety signalling than VA chemoreceptors [15]. However, there is a multitude of evidence to suggest that small intestinal VAs act as nutrient sensors, responding to gut hormones released from EECs upon exposure to nutrients to induce satiety.

Within the small intestine gut-hormone release is region and nutrient specific. For example, the gastrointestinal hormone CCK is released from I-cells, primarily in the duodenum and proximal jejunum, in response to luminal fatty acids and proteins [47,48,49]. In contrast, peptide YY (PYY) and GLP-1 are predominantly released from L-cells in the ileum and in humans distal (190 cm from the pylorus) glucose infusion substantially increased plasma GLP-1 levels, whereas proximal infusion (13 cm beyond the pylorus) had minimal effect on GLP-1 levels in healthy individuals and type 2 diabetic patients [50]. However, despite this PYY and GLP-1 have been observed in the porcine duodenum and jejunum suggesting a more global role for these hormones throughout the small intestine [51] and a study utilizing equicaloric intra-duodenal infusion of glucose and intralipid showed that fat is more potent at stimulating GLP-1 secretion compared to glucose in healthy males [52]. Co-expression of gut hormones, originally considered to be synthesized in distinct populations of EECs such as I-cells or L-cells, has been observed suggesting EECs are a single cell type which can produce an array of peptides depending on the location and environment [53]. PYY and GLP-1 are released in response to carbohydrates, fatty acids and amino acids. All these hormones have established anorexigenic effects and given their short plasma half-life, particularly for GLP-1, a local paracrine action is more probable than an endocrine action [54]. CCK has pronounced effects on food intake, reducing meal size and cumulative food intake [55]. The majority of the effects of CCK on food intake are due to action at CCKA receptors expressed on VAs [56,57]. PYY knockout mice are hyperphagic and exhibit a delayed satiety response to luminal nutrients, implying a role in the regulation of energy balance [58], whereas analogues of PYY, such as PYY (3–36), inhibit food intake [59]. Further, PYY receptors (Y2R) are expressed in VA neurons [60] and exogenous administration of PYY (3–36) increases VA firing [61]. In addition, bilateral subdiaphragmatic vagotomy led to the loss of PYYs anorexigenic effects [62]. However, treatment with capsaicin, to destroy VA fibres, had no effect on the inhibitory effect of PYY on food intake [63]. This could be due to non-specific effects on other fibre types [64] or the incomplete lesion induced using capsaicin [63]. The gut hormone GLP-1 has also been shown to increase satiation and reduce food intake. These effects are likely mediated via VAs as capsaicin treatment [63], bilateral sub-diaphragmatic vagotomy [62], and vagal deafferentation [65] resulted in loss of the anorexigenic effects of GLP-1 and its analogues. Further, GLP-1 receptors are expressed in rat VA neurons [66] and selective knockout of these receptors was associated with increased meal size, however, there was no long-term effects on energy balance [67]. Overall, it is likely that nutrients acting at specific sites on EECs initiate an intracellular process resulting in the release of a peptide/hormone, such as GLP-1, CCK, or PYY. Subsequently these hormones activate VA endings which send signals to the brain where it is processed leading to feelings of satiety and fullness, ultimately terminating a meal.

### 2.3. Plasticity of Gastrointestinal Afferents

Gastrointestinal VAs demonstrate a high degree of plasticity in order to precision match food intake to energy requirements. For example, after a fasting period energy demand is high and the first meal is increased, specifically the size and duration of the meal [68,69]. In fact, the size of this first meal has been shown to be directly associated with the length of the fasting period [69]. Further, it is known that gastric VA tension receptor responses to stretch are dampened after a fast [70]. Thus, more food would need to enter the stomach to signal the same response elicited in the fed state. This may, at least in part, explain why the first meal after a fast is increased compared to control. Whether the reduced mechanosensitivity depends on the length of the fasting period remains to be determined. A more chronic physiological adaptation occurs during pregnancy. During pregnancy gastric VA responses to stretch, assessed during the light phase, were attenuated in mice [71]. This is accompanied by an increase in food intake, predominantly due to an increase in meal size, during the light but not the dark phase and thus appears to be circadian in nature [71]. It is known that gastric VAs display diurnal rhythms in their response to food related stimuli [2], to regulate food intake over a 24 h period. The pregnancy data suggest that adjustments in the diurnal sensitivity of gastric VAs might play an important role in the fine tuning of food intake over longer periods of time, such as during pregnancy. However, this is speculative and requires further investigation. Nonetheless, diurnal rhythms in gastric VA sensitivity to food related stimuli is another example of the plasticity of VAs and this will be discussed in the next section. 

## 3. Circadian Regulation of Food Intake

### 3.1. Circadian System and Food Intake Patterns

The natural feeding behaviour in most living organisms is to spend one phase of the light-dark cycle in an active and feeding state and the other in a resting and fasting state [72]. For instance, humans and other diurnal mammals naturally spend the light phase in the active and feeding state, whereas, nocturnal mammals, such as rodents, are generally active and feeding during the dark phase. For example, ad libitum standard chow fed mice consume between 65 and 80% of food during the dark phase [3,73,74,75].

These rhythms are controlled by the circadian clock system which has a central clock located in the hypothalamic suprachiasmatic nucleus (SCN). The SCN influences other clocks located both centrally and peripherally [76] to temporally regulate metabolic processes over a 24 h period, giving rise to circadian rhythms in energy expenditure [77,78] and appetite/hunger [79,80,81,82]. In turn, the SCN is regulated by the light-dark cycle, through information received from the retina via the retinohypothalamic tract [5]. The molecular mechanisms driving circadian oscillations in the SCN consist of a series of interlocking transcriptional-translational molecular feedback loops. Briefly, clock genes consist of positive and negative elements. Positive elements include Circadian Locomotor Output Cycles Kaput (Clock), Brain and Muscle ARNT-Like 1 (BMAL1) and neuronal PAS domain protein 2 (NPAS2). Heterodimers of these positive elements, including BMAL1/Clock or BMAL1/NPAS2, enter the nucleus and stimulate the transcription of negative elements, such as period 1, 2 and 3 (Per1–3) and cryptochrome 1 and 2 (Cry1–2) [83,84,85]. In turn, the protein products of these transcriptional factors translocate to the nucleus and inhibit the activity of the BMAL1/Clock complex, ultimately inhibiting their own transcription and allowing the build up of the BMAL1/Clock complex to initiate a new cycle. In addition, there are also numerous nuclear receptors, including REV-ERB and ROR, which are considered to be core components of the clock system [86]. The heterodimer BMAL1/Clock has been shown to activate the transcription of REV-ERBα, which subsequently represses the transcription of BMAL1 [87,88]. These nuclear receptors enable bi-directional communication between the circadian system and other physiological systems and allows clock rhythms to be influenced by, for example, hormonal signals and cellular redox status [89,90,91]. 

Data using genetically modified mice with knockout or mutated clock genes provide compelling evidence for the role of clock molecules in the regulation of diurnal rhythms in food intake. For example, diurnal feeding rhythms in homozygous Clock mutant mice are greatly attenuated and the mice are also hyperphagic [92,93]. Similarly, loss or attenuation of feeding rhythms and/or hyperphagia has been observed in other clock gene mutant mice, such as BMAL1 [94], Cry1–2 [95,96] and Per2 mutant mice. 

There are connections between the SCN and other regions in the hypothalamus and beyond involved in energy homeostasis, including but not limited to the arcuate nucleus (ARC), dorsal medial hypothalamus (DMH) and paraventricular nucleus [97,98,99,100]. These connections are essential for the day-to-day organization of physiological rhythms, such as food intake and energy expenditure. In addition to receiving projections and information from the SCN, many sites within the hypothalamus, including the ARC and DMH, possess their own circadian oscillators [101]. This has been demonstrated in cultured brain slices from PER2::Luciferase reporter mice and in long-term electrophysiological recordings [102]. The ARC and DMH have established roles in food intake regulation [103] and both regions are critical in driving diurnal rhythms in feeding behaviour. For instance, there are diurnal rhythms in the expression of ARC neuropeptides involved in food intake regulation, such as neuropeptide Y (NPY) [104], pro-opiomelanocortin (POMC) [105], and cocaine and amphetamine regulated transcript (CART) [105,106]. In rats, targeted destruction of ARC leptin- or NPY-responsive neurons, using saponin conjugated ligands, resulted in a disruption of the diurnal feeding rhythms [107,108]. In addition, deletion of the NPY receptors Y2 and Y4 altered daily feeding patterns [109]. In contrast, selective deletion of POMC neurons had no effect on diurnal rhythms in feeding behaviour in mice [110], although altered feeding rhythms were observed in Per2 knockout mice and linked to disruption in the diurnal rhythms of α-melanocyte-stimulating hormone, a POMC cleavage product [111]. Other central sites are involved in the control of feeding behaviour, however, there are numerous reviews on this area [100,112] and the focus of this review is gastrointestinal VAs.

Similar clock oscillators are located in peripheral tissue, including the gastrointestinal tract, adipose tissue and muscle, and the SCN sends neural or hormonal signals to these clocks to align and prevent the dampening of these rhythms [113]. For example, the neural links, e.g., VAs, between the gut and the central nervous system have also been shown to contain clock oscillators [2]. 

### 3.2. Circadian Vagal Afferent Signalling

**Figure 2:**
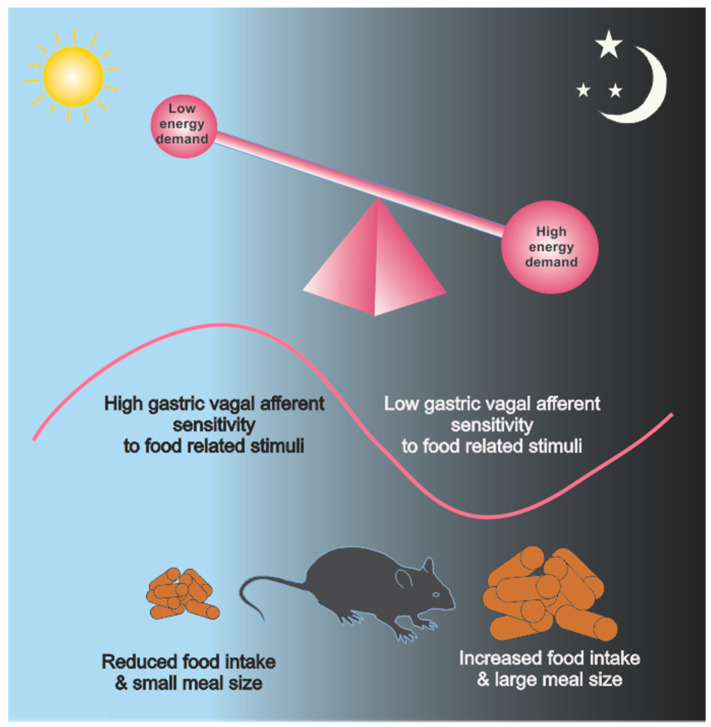
Schematic of the relationship between energy demand (e.g., during the active dark phase (dark grey region) verses inactive light phase (light blue region)), gastric vagal afferent (VA) sensitivity to food related stimuli (pink line) and food intake and meal size in mice. During the light phase the mice are resting and energy demand is low. Further, gastric VA sensitivity is high and associated with reduced food intake and meal size. Conversely, during the dark phase mice are active and consequently energy demand is high. In addition, gastric VA sensitivity is low and associated with increased food intake and meal size.

The diurnal sensitivity of gastric VAs to food related stimuli is a good example of the high degree of plasticity these afferents exhibit (Figure 2). Gastric VA mucosal and tension receptors display diurnal rhythms in their response to mucosal stroking, using calibrated von Frey hairs, and stretch, respectively, with peak responses during the light phase and a nadir during the dark phase in mice [2]. In addition, these oscillations in gastric VA mechanosensitivity are inversely associated with the amount of food present in the stomach [2]. Meal size varies considerably between the light and dark phase in rodents, with larger and more frequent meals in the active dark phase when energy requirements are high [114]. Activation of VA tension receptors, by gastric distension has been shown to induce satiety [39] and, therefore, reduced sensitivity of gastric tension receptors, during the active dark phase, would permit more food to be consumed before satiation is reached. Whilst this is the only report of gastrointestinal VA circadian rhythmicity it has been shown that colonic afferents also exhibit circadian variation in their response to mechanical stimulation [115]. In this study it was observed that the spinal afferent mediated pain response to colonic distension was greater during the dark than the light phase in rats [115].

Although gastric VAs are predominantly mechanosensitive, the response of gastric VAs to food related stimuli can be modulated by peptides and hormones, including gastric hormones such as the ‘hunger hormone’ ghrelin. Ghrelin receptors are expressed in VA cell bodies and ghrelin attenuates gastric VA responses to mechanical stimuli. Of particular significance are the nutritional status dependent inhibitory effects of ghrelin on mucosal receptor mechanosensitivity; with inhibition observed in fasted mice but not *ad libitum* fed mice [70]. However, over a 24 h period, despite the distinct feed-fast episodes, the inhibitory effect of ghrelin on mouse gastric VAs did not display diurnal rhythms [3], suggesting a prolonged fast is required to elicit these effects. In addition, ghrelin plasma levels display circadian rhythms with a peak and trough during the inactive and active phases, respectively, in rodents and humans [116,117], therefore, it is unlikely to be mediating diurnal rhythms in gastric VA mechanosensitivity, as peak gastric VA sensitivity (Figure 2) occurs at about the same time as peak ghrelin levels in mice. 

As stated earlier, gastrointestinal VA signals impact on the reflex control of gut motility. Although the focus of this review is food intake, it is important to recognize that changes in gut motility may be a consequence of changes in VA sensitivity. For example, it has been shown that gastric emptying half-time, of a solid not liquid meal, in humans is significantly longer in the evening (8 p.m.) compared to a morning (8 a.m.) meal [118]. This could be due to the diurnal sensitivity of gastric VAs responses to the meal which would presumably lead to diurnal reflex control of gastric emptying, however, this requires further investigation.

#### 3.2.1. Nutrient and Gut Hormone Signals

In the gastrointestinal tract other mechanisms involved in gut to brain signalling via VAs may be responsible for circadian regulation of satiety signals, such as the nutrient sensing mechanisms within the intestine. For example, T1R2, a component of the sweet taste receptor, displayed circadian rhythmicity with peak expression in the mouse proximal jejunum, just prior to the commencement of the dark phase [119]. In theory, as these sweet taste receptors are co-localised with gut hormones, such as GLP-1, in EECs within the small intestine [120,121] and carbohydrates (e.g., glucose and sucrose) stimulate secretion of GLP-1 from EEC lines [121], diurnal rhythms in sweet taste receptors should lead to diurnal rhythms in GLP-1 secretion in response to the same nutrient load. However, there was no apparent difference in GLP-1 or PYY secretion, in response to a standard liquid Ensure meal given in the middle of the dark or light phase in rats [122], which is consistent with the lack of diurnal rhythms in T1R2 in the rat jejunum [123]. Further, research is required to clarify the role if any of the circadian system in gastrointestinal nutrient sensing and possible species variations.

#### 3.2.2. Gut Microbiota

The gut microbiota may also play a role in regulating diurnal rhythms in gastrointestinal VA function. Bacterial-derived molecules can influence VA function either directly or indirectly via activation of receptors on EECs (see review [124]). For example, receptors for short-chain fatty acids (SCFAs), produced by the gut microbiota, are present on EECs and the SCFA propionate has been shown to stimulate the release of PYY and GLP-1, both in vitro and in vivo in humans [125,126,127], which can subsequently activate intestinal VAs. Up to 60% of total microbial composition oscillates over a 24 h period, translating to 20% of commensal species in the mouse and 10% in humans [128]. In addition, there are daily rhythms in SCFA production [129], which is dependent on the timing of food intake and the supply of substrate (dietary fibre) for microbiota production of SCFAs. Therefore, it is possible a disruption in food intake patterns, such as occurs in shift workers, would lead to disruption in SCFA production which could conceivably lead to disruption in gastrointestinal VA satiety signalling that would further disrupt food intake patterns, perpetuating the situation. However, this is highly speculative and requires more detailed investigation. The host circadian clock has also been shown to regulate microbiota production of SCFAs, with disruption of SCFA production observed in Bmal knockout mice [130]. However, this is restored by dark-phase time restricted feeding, suggesting that disruption of SCFA production in Bmal knockout mice is secondary to changes in the timing of food intake. 

#### 3.2.3. Disrupted Circadian Signalling

##### High Fat Diet-Induced Obesity

**Figure 3:**
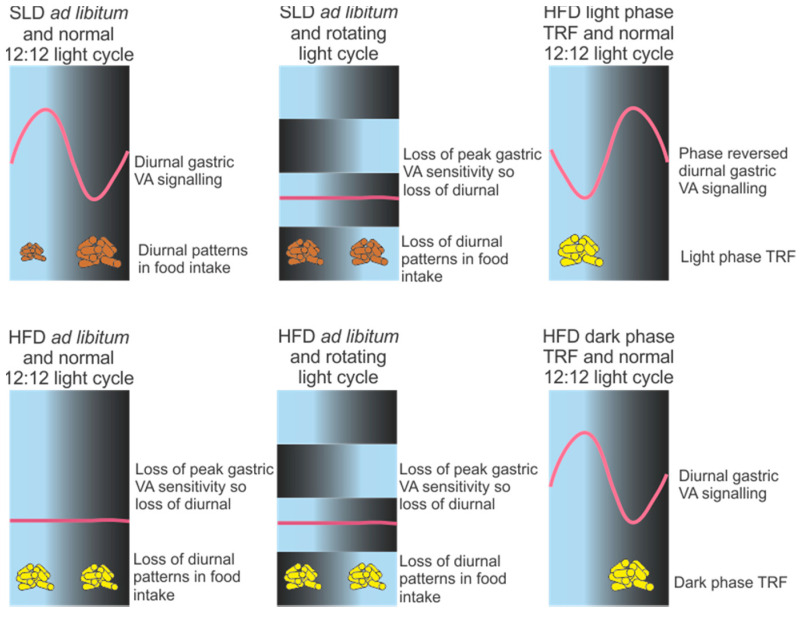
Schematic of the effect of circadian desynchrony on diurnal gastric vagal afferent (VA) responses to food related stimuli (e.g., stretch or mucosal stroking) in mice. On a normal standard laboratory diet (SLD) gastric VAs display diurnal rhythms in sensitivity to food related stimuli, with associated diurnal rhythms in food intake. In high fat diet-induced obese mice and/or mice exposed to a rotating light cycle diurnal rhythms in gastric VA responses to food related stimuli are lost and associated with a disruption in diurnal food intake patterns. High fat diet-fed mice exposed to a time restricted feeding (TRF) protocol, where food is restricted to the 12 h light phase or 12 h dark phase, retain diurnal rhythms in gastric VA responses to food related stimuli.

Disruption of the diurnal rhythms in gastric VA satiety signalling may impact on food intake and contribute to metabolic disorders. In high fat diet-induced obese mice the diurnal rhythm in gastric VA satiety signalling was lost and associated with a loss or attenuation in the diurnal patterns of food intake [3] (Figure 3). For example, in high fat diet-induced obese mice there was an increase in meal size during the light phase, to levels equivalent to meal size in the dark phase [3]. This loss of diurnal rhythms in gastric VA mechanosensitivity is probably due to the obese state rather than the high fat diet as circadian rhythms in gastric VA mechanosensitivity were still observed two and four weeks after commencement of the high fat diet, at stages when there is no significant difference or the point of transition to increased weight gain, respectively, compared to standard chow fed mice [3,131]. The loss of diurnal rhythms in high fat diet-induced obese mice is predominantly due to a loss of the peak gastric VA mechanosensitivity observed during the light phase [2], allowing more food to be consumed when mice are inactive and energy demand is low. Jejunal VA responses to distension and chemical (e.g., CCK) stimuli are also attenuated during the light phase in high fat diet-induced obese mice [132] and, therefore, it is possible that jejunal VAs have a similar circadian profile as gastric VAs, although to date this remains to be determined. Further, the attenuated response, observed during the light phase, does not return to normal upon return to a standard chow diet for an equivalent time on the high fat diet [133]. This reduction in gastric VA tension receptor mechanosensitivity is consistent with the observed reduction in neural activation, in response to gastric distension, within the hypothalamus of obese individuals [134]. In addition, the failure of gastric VAs to revert back to the lean phenotype has also been observed in the neuronal responses to food intake in the brain of post-obese individuals [135]. It is acknowledged that chronic feeding of an energy dense and palatable diet leads to obesity [136], which once established is defended against weight loss [137] or weight perturbations [138]. It is possible the reduction in gastric VA responses to distension may contribute to the difficulty in maintaining weight loss.

##### Disrupted Light Cycle

Long-term misalignment of the circadian system, such as occurs in shift work, is a risk factor for metabolic disorders, including obesity [139,140,141,142]. This is a huge problem as ~15–20% of the working population are shift workers, including those on permanent night shifts or on rotating or irregular schedules [143,144,145]. As stated previously, animals and humans normally exhibit diurnal rhythms in food intake with the majority of food consumed during the natural active/wake period. These daily rhythms in food intake are aligned with other zeitgebers, such as the light cycle and activity, where they can act synergistically to promote synchronization of daily rhythms, including appetite, anabolism and catabolism. Misalignment of these zeitgebers can occur for a number of reasons, including shift work, exposure to long hours of artificial light and even dim light during the dark phase, and result in disruption of the syncrony of daily physiological and behavioural rhythms. For example, shift workers who are active during the rest period and exposed to long hours of artificial light also eat meals around their working hours [146,147,148,149] resulting in increased food intake during a period when they normally rest. Interestingly, although total energy intake is similar between night and day shift workers [150], the pattern of food intake is altered with night shift workers spreading food intake across a 24 h period with no extended fasting periods [151]. In addition, shift workers usually revert back to a more social daytime schedule on their days off, imposing a jet-lag model as physiological processes attempt to adjust to the new schedule. Similarly, the ready availability of artificial light has extended the length of the day period which has, subsequently, led to an extension of the feeding period. For example, a smart-phone application designed to monitor food intake has been used to demonstrate that feeding episodes span over 15 h/day in >50% of participants [152].

There are a number of animal models of shift work, including alterations in activity and/or sleep or light exposure [153], which have shown that circadian misalignment promotes metabolic disturbances, such as obesity [153,154,155]. For example, weight gain was higher in mice exposed to dim light at night compared to mice exposed to a normal light-dark cycle and, despite no change in overall 24 h food intake, there was disrupted feeding patterns due to increased light phase food intake [156,157,158]. Further, in a mouse rotating light cycle model of shift work, diurnal rhythms in gastric VA sensitivity to food related stimuli was ablated and accompanied by disruptions in diurnal food intake patterns [159]. In this study the lean standard laboratory and obese high fat diet-fed mice exposed to the rotating light cycle gained more weight than their counterparts on a normal 12 h light-12 h dark cycle, despite the fact there was no overall change in 24 h energy intake [159]. Therefore, diurnal gastric VA mechanosensitivity is susceptible to disturbances in the light-dark cycle and the obese state. These disturbances are associated with changes in food intake patterns and likely contribute to the difficulty in losing and maintaining weight loss. It is unclear whether the disruption in diurnal rhythms in VA sensitivity is causing the disruption in food intake or vice versa but, in spite of this, time restricted protocols to re-establish fed-fasting regimes is an attractive option to reinforce circadian rhythms.

#### 3.2.4. Time Restricted Feeding

The timing of food intake episodes is another important regulator for central and peripheral clocks [160]. Time restricted feeding, where food intake is limited to a specific number of hours per day (6–12 h), provides a mechanism to re-establish diurnal rhythms in fed/fasting states, realigning and reinforcing circadian rhythms. Time restricted feeding has been shown to protect against metabolic disease in high fat diet-fed mice without reducing energy intake [161]. Further, time restricted feeding prevented obesity in mouse models of jetlag or shift work [162]. The metabolic benefits of time restricted feeding have been reviewed extensively [163] and, therefore, this review will focus on gastrointestinal VA satiety signalling and the impact of time restricted feeding on these signals. Early light phase time restricted feeding (800–1400 h), in humans, reduced appetite and, perhaps more important in terms of gastrointestinal VA function, increased feelings of fullness [164]. Twelve-hour time-restricted feeding in the light or dark phase prevented the loss of diurnal rhythms in gastric VA satiety signalling in high fat diet-induced obese mice, albeit the rhythms were phase reversed when the feeding was restricted to the light phase [131]. It remains to be determined whether time restricted feeding will reverse the loss of rhythms in VA sensitivity in established high fat diet-induced obesity [3] or shift work conditions [159] and, therefore, whether time restricted feeding is a potential solution to the lack of reversal of these afferents, to the pre-obese diurnal phenotype, upon return to a normal diet in mice [133]. Further, it remains to be established whether a time restricted feeding protocol is only required for a short period to switch the VA phenotype back to the pre-obese state or whether there needs to be a maintenance protocol, such as time restricted feeding for 3–4 days per week.

## 4. Conclusions

Gastrointestinal VAs play an essential role in the short-term regulation of food intake. The sensitivity of these afferents is not static, displaying diurnal sensitivity to food related stimuli in order to finely control food intake, over a 24 h period, to match the daily fluctuations in energy demand. Disruption of these diurnal rhythms, such as occurs in diet-induced obesity and shift work, is associated with disrupted food intake patterns which, without increases in energy intake, can lead to weight gain due to misalignment of metabolic processes with food intake. In diet-induced obesity the loss of these rhythms can be prevented using a time restricted feeding protocol. It remains to be determined whether time restricted feeding will reverse the loss of diurnal signals in established diet-induced obesity.
